# Lymphoma of the orbit masquerading as Tolosa-Hunt syndrome

**DOI:** 10.1186/s12886-015-0037-8

**Published:** 2015-05-15

**Authors:** Tarek A Shazly, Ellen B Mitchell, Gabrielle R Bonhomme, Joel S Schuman

**Affiliations:** UPMC Eye Center, Department of Ophthalmology, University of Pittsburgh Medical Center, Pittsburgh, PA USA; 203 Lothrop St, Pittsburgh, PA 15213 USA

**Keywords:** Lymphoma, Tolosa-Hunt, Neuro-imaging, Cavernous sinus, MRI

## Abstract

**Background:**

Tolosa-Hunt syndrome is a rare clinical syndrome characterized by painful ophthalmoplegia and ipsilateral cranial neuropathies. It is caused by an inflammatory process of unknown etiology.

**Case presentation:**

We present a case of a 77-year-old white man with history of Waldenstrom’s macroglobulinemia transforming to large B-cell lymphoma who presented to a community physician complaining of 4 months of isolated right retro-orbital pain and later with diplopia, ptosis, 6th nerve and pupil-sparing partial 3rd nerve palsies as well as progressive neurological findings. His clinical course was complicated by debilitating neurological symptoms and multiple hospitalizations leading to a delay in diagnosis caused by incomplete initial workup.

**Conclusion:**

This case is a reminder that lymphoproliferative disorders often mimic other neurologic disorders and that Tolosa-Hunt is a rare diagnosis that must be considered a diagnosis of exclusion.

## Background

Tolosa-Hunt syndrome (THS) is a rare clinical syndrome characterized by painful ophthalmoplegia and ipsilateral cranial neuropathies [1-7]. It is caused by an idiopathic granulomatous inflammation of the cavernous sinus or orbital apex complex that is exquisitely responsive to parentral glucocorticoids. The estimated annual incidence is one case per million per year in the US [3]. This rare clinical entity was first described in 1954 as a syndrome comprised of two features: (a) pain in the first division of the trigeminal nerve; and (b) progressive paralysis, partial or total, of the oculomotor nerve and occasionally of the fourth, sixth, and the fifth cranial nerves [1]. Tolosa-Hunt syndrome is a diagnosis of exclusion and must be carefully differentiated from life threatening diagnoses, a mandate challenged by the lack of a specific diagnostic test to identify this disorder [3].

Tolosa-Hunt syndrome is caused by an inflammatory process of unknown etiology [4]. The inflammation leads to compression and secondary dysfunction of the structures within the cavernous sinus, including cranial nerves III, IV, and VI, as well as the ophthalmic and maxillary divisions of the trigeminal nerve resulting in clinical symptoms [1-7].

## Case presentation

A cachectic 77 year-old white male with history of Waldenstrom’s macroglobulinemia transforming to large B-cell lymphoma presented with right eye pain for 4 months without any visual symptoms. He is an ex-smoker with 0.5 packs per day over 30 years of tobacco smoking (quit in 2003). He currently is retired and lives with his wife. He denies consuming alcohol or controlled substances.

On day 1 of his symptoms he presented to a community physician with right retro-orbital pain. A Magnetic Resonance Imaging (MRI) scan of the brain with IV contrast was ordered given his history of lymphoma. His MRI was negative for acute pathology. Given his negative scan and the severity of pain, he was treated with oral valacyclovir along with prednisone for presumed right Herpes Zoster ophthalmicus.

One week later, he presented urgently to a local emergency room for a new onset of diplopia and right eyelid ptosis. His examination was significant for a right partial, pupil involving third nerve palsy. A repeat MRI of the brain and orbits with IV contrast was obtained, and revealed right lacrimal gland enlargement. A lumbar puncture (LP) was performed, and CSF analysis was negative for abnormal cells, leading to a presumptive diagnosis of right sided Tolosa Hunt Syndrome. He received the presumptive diagnosis of right sided Tolosa-Hunt Syndrome. He received a dose of 500 mg of intravenous (IV) methylprednisolone in the emergency room which achieved a prompt and significant reduction in his pain level. His blood sugar level became elevated in response to the IV steroids. He was discharged to home with the instructions to use 60 mg of oral prednisone and an insulin sliding scale until he was seen by his primary care physician.

Four weeks later, he saw his local ophthalmologist for persistent diplopia despite complete resolution of his pain and ptosis. At that time, he was on 10 mg/day of prednisone. He had developed severe, progressive muscle weakness due to steroid induced myopathy and was confined to a wheelchair. He then underwent surveillance Positron Emission Tomography – Computed Tomography (PET-CT) for lymphoma. The scan incidentally revealed asymptomatic perforated diverticulitis. He was urgently admitted to the hospital and had a prolonged hospital course with conservative treatment, including IV Piperacillin/Tazobactam, nil per os (NPO) and gradual Prednisone taper, with discharge to home on Prednisone 5 mg/day.

Less than a week later he developed a new non-productive cough. Chest computed tomography (CT) revealed interval lung consolidation. Lung biopsy was performed and revealed Pneumocystis carinii for which he received a 3 week course of atovaquone.

Four weeks later (Week 10 of symptoms), he was referred to the Neuro-ophthalmology division for evaluation of persistent 3rd cranial nerve palsy with negative neuroimaging. At that time his only ocular symptom was binocular diplopia. On examination his visual acuity and color vision were normal in each eye, with full Humphrey visual fields. His motility exam was significant for bilateral 6^th^ nerve palsies and left partial pupil sparing 3^rd^ nerve palsy. Given the high suspicion for lymphomatous infiltration despite the negative prior neuroimaging, a repeat brain and orbit MRI as well as LP were obtained and were both unrevealing.

The case was extensively discussed with the patient’s oncologist, local ophthalmologist and neurologist, however, they opted to continue present management. He was then lost to follow up for 7 weeks before his return visit with the complaints of unchanged diplopia and new right sided facial dysthesia, worsened right ptosis and return of his his right eye pain. His exam now revealed a right pupil involving 3^rd^ nerve palsy, right facial palsy, and sensation was decreased over right V1, V2 and V3. Given the progression of his cranial neuropathies, he was admitted for repeat brain and orbit MRI, LP, and CT Angiography of the head and neck. These tests were initially interpreted as unrevealing.

**Figure 1:**
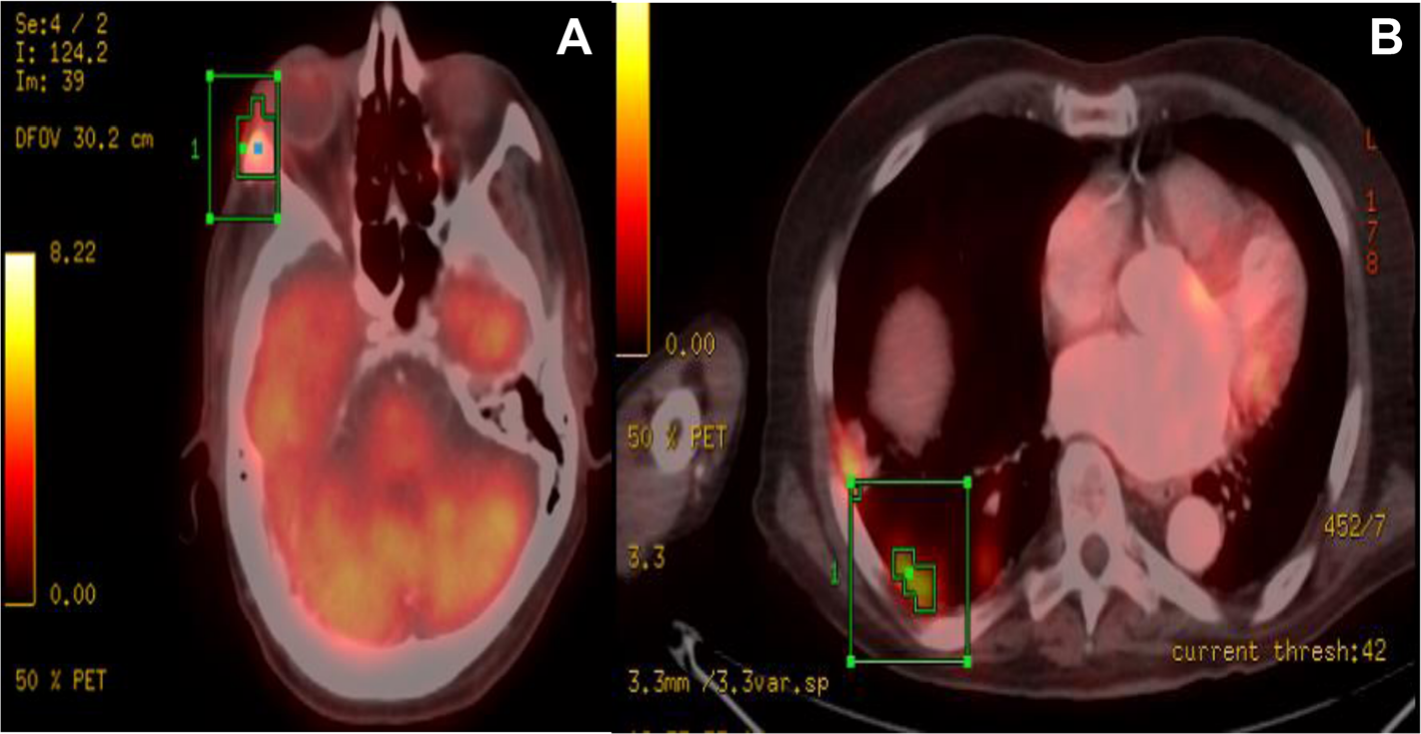
PET-CT Contrast-enhanced helical CT was performed and PET emission data 1 hour following IV injection of 18.1 mCi of F-18 FDG. **A**: Shows a new focus of abnormal metabolic activity at the lateral wall the right orbit with underlying sclerosis. Maximal SUV value is 4.6. **B**: Shows area of dense consolidation with high metabolic activity suggestive of malignancy rather than an infection or inflammation. Additionally, multiple hot spots involving the R. parotid, mandibular angle, lateral wall the R. orbit, sternum, both humeri and femurs were detectable.

**Figure 2:**
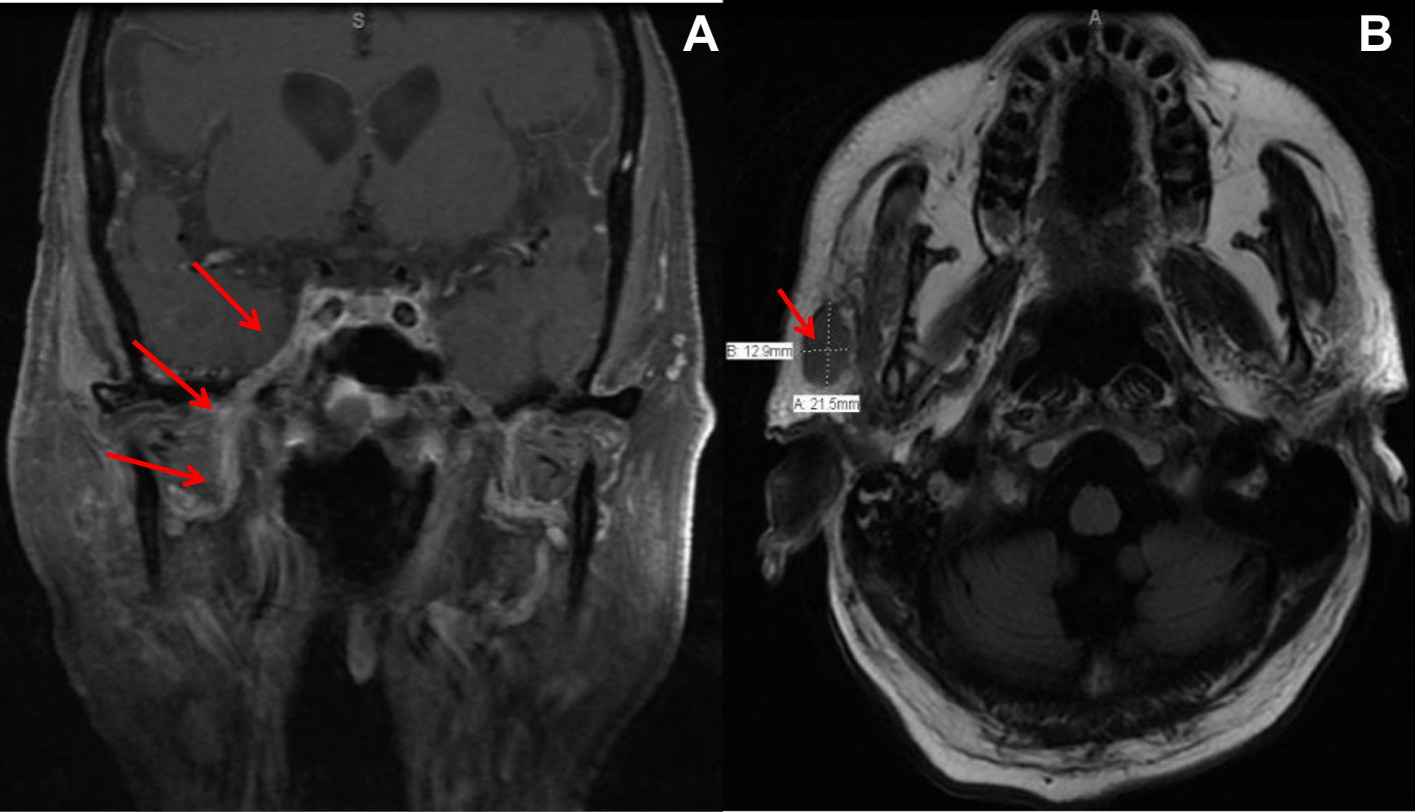
MRI Brain and orbit with and without contrast. **A**: asymmetric enhancement in the right Meckels cave along the V3 division of the trigeminal nerve suggestive of perineural spread of tumor. **B**: overlying the right parotid gland, there is a new 22 × 13 mm heterogeneously enhancing lymph node.

Two weeks later, his routine PET/CT revealed a new focus of abnormal metabolic activity involving the lateral wall the right orbit, with underlying sclerosis. It also revealed an area of dense lung consolidation with high metabolic activity suggestive of malignancy (Figure 1). Additionally, multiple hot spots involving the right parotid, mandibular angle, lateral wall the sternum, both humeri and femurs were detected. An urgent MRI of the brain and orbit revealed asymmetric enhancement involving Meckel’s cave along the V3 division of the right trigeminal nerve suggestive of peri-neural spread of tumor (Figure 2). Overlying the right parotid gland, there was a new 22 × 13 mm heterogeneously enhancing lymph node. An enhancing, bone marrow-replacing lesion within the left aspect of the clivus, with involvement of the right aspect of the sphenoid floor, and lesions adjacent to the right carotid canal.

Parotid fine needle aspiration biopsy revealed diffuse large B-cell non-Hodgkin lymphoma with high-grade features. Chemotherapy with Gemcitabine, Dexamethasone, and Rituximab was initiated along with 3D conformal radiation of the orbit with good response.

Five months later, his diplopia and ocular motility were slightly improved. He developed exposure keratopathy with stable visual acuity and no evidence of radiation retinopathy. He was then referred to adult strabismus service for definitive treatment of his residual binocular diplopia.

### Discussion

In 1988, the International Headache Society defined the diagnostic criteria of THS to include; episode(s) of unilateral orbital pain for an average of 8 weeks if untreated, with associated paresis of one or more of the third, fourth, and sixth cranial nerves. Cranial nerve paresis may coincide with the onset of pain or follow it within a period of up to 2 weeks, and the pain must be relieved within 72 hours of initiation of corticosteroid therapy. Other causative space-occupying or infiltrative lesions must be excluded by neuro-imaging. Förderreuther *et al.* reported 6 cases were the aforementioned criteria mis-lead to the diagnosis of THS in the presence of other pathology, and recommended revising the criteria. In his series, the 6 patients were found to have a parasellar chondrosarcoma, inconclusive pathological exam of cavernous sinus mass due to small sample, chordoma extending into the cavernous sinus, suspected meningioma versus THS, diabetic microvascular third nerve palsy, and cerebral vasculitis. He included “Other causative lesions must be excluded by neuro-imaging, especially of the region of the cavernous sinus and the orbita, and by blood and cerebrospinal fluid (CSF) examinations” [8]. They also recommended that clinical and radiological follow-up examinations must be performed for at least 2 years, even in patients with negative findings on magnetic resonance imaging at onset.

Orbital lymphomas are relatively rare, comprising only 1% of all non-Hodgkin’s lymphoma [9]. However, orbital lymphomas are the most common primary orbital tumor in adults 60 years of age and older [10]. Margo and Mulla reported a 55% rate of lymphomas involving the orbit amongst 300 patients with orbital malignancies [11]. Eckardt et al. reported diagnostic delay in patients with orbital lymphoma due to the non-specific presentation [12]. Once diagnosis is established and staging is complete, radiation therapy is the recommended treatment for stage IEA patients. Systemic chemotherapy is indicated in selected stage IIEA patients and in patients with stage IIIEA disease [12].

Our patient was started on corticosteroids for two different presumptive diagnoses: Herpes Zoster Ophthalmicus and THS. The prolonged steroid course caused a number of complications, including myopathy, pneumonia, and hyperglycemia, and delayed treatment of his underlying malignancy. This case is a reminder that Tolosa-Hunt Syndrome (THS) is a rare disorder and that it must remain a diagnosis of exclusion. Life or vision-threatening conditions may mimic THS. These conditions should be carefully excluded prior to considering empiric corticosteroid therapy. It should also be remembered that corticosteroid not only improves the signs and symptoms of THS but may mask a number of neoplastic, inflammatory and lymphoproliferative disorders, delaying definitive treatment or diagnosis [8].

Additionally, any diagnosis of THS should be challenged when clinical worsening occurs after parenteral steroids.

## Conclusion

A high index of suspicion is indicated to exclude neoplasia and any lymphoproliferative disorder in high risk patients with evolving multiple cranial neuropathies. New or evolving findings on successive clinical exams, particularly after treatment with parenteral steroids, should prompt neuroimaging and extensive diagnostic testing to exclude malignancy or a space-occupying lesion mimicking THS. Tolosa-Hunt should remain a diagnosis of exclusion.

## Consent

Written informed consent was obtained from the patient for publication of this case report and any accompanying images. A copy of the written consent is available for review by the Editor of this journal.

## Abbreviations

THS: Tolosa-Hunt syndrome
CHOP-R: Cyclophosphamide, hydroxydaunorubicin, oncovin, prednisone – rituximab
MRI: Magnetic Resonance Imaging
LP: Lumbar puncture
IV: Intravenous
PET-CT: Positron Emission Tomography – Computed Tomography
NPO: Nil per os
CT: Computed tomography
CSF: Cerebrospinal fluid
